# Experimental evidence of the synergistic effects of warming and invasive algae on a temperate reef-builder coral

**DOI:** 10.1038/srep18635

**Published:** 2015-12-22

**Authors:** Diego K Kersting, Emma Cebrian, Clara Casado, Núria Teixidó, Joaquim Garrabou, Cristina Linares

**Affiliations:** 1 Departament d’Ecologia. Facultat de Biologia. Universitat de Barcelona (UB), 08028 Barcelona, Spain; 2 Centre d’Estudis Avançats de Blanes (CSIC), 17300 Girona, Spain; 3 Departament de Ciències Ambientals. Facultat de Ciències. Universitat de Girona, 17004 Girona, Spain; 4 Institut Ciències del Mar (ICM-CSIC), 08003 Barcelona, Spain; 5 Stazione Zoologica Anton Dohrn, 80121 Naples, Italy; 6 Aix-Marseille University, Mediterranean Institute of Oceanography (MIO), 13288, Marseille, Cedex 9; Université de Toulon, 83957, CNRS/IRD, France

## Abstract

In the current global climate change scenario, stressors overlap in space and time, and knowledge on the effects of their interaction is highly needed to understand and predict the response and resilience of organisms. Corals, among many other benthic organisms, are affected by an increasing number of global change-related stressors including warming and invasive species. In this study, the cumulative effects between warming and invasive algae were experimentally assessed on the temperate reef-builder coral *Cladocora caespitosa*. We first investigated the potential local adaptation to thermal stress in two distant populations subjected to contrasting thermal and necrosis histories. No significant differences were found between populations. Colonies from both populations suffered no necrosis after long-term exposure to temperatures up to 29 °C. Second, we tested the effects of the interaction of both warming and the presence of invasive algae. The combined exposure triggered critical synergistic effects on photosynthetic efficiency and tissue necrosis. At the end of the experiment, over 90% of the colonies subjected to warming and invasive algae showed signs of necrosis. The results are of particular concern when considering the predicted increase of extreme climatic events and the spread of invasive species in the Mediterranean and other seas in the future.

Global change-related disturbances, including warming and the spread of invasive species, are affecting marine ecosystems worldwide^1^,^2^. The vast geographical range in which these stressors act has resulted in an overlap in their occurrence in many geographic areas, especially in global change hot spots such as the Mediterranean Sea^3^,^4^,^5^,^6^. Knowledge of the interaction of stressors, and the resulting cumulative impacts, is essential to understand and predict the response and potential resilience of benthic organisms^7^,^8^,^9^,^10^. However, while multiple stressor’s interactions have been studied in coral reefs^10^, to our knowledge, the interaction of warming and invasive species has not been considered yet, despite the increasing concurrence and future predictions.

Scleractinian reef-builders have been critically impacted by warming in tropical^11^,^12^ and temperate seas^5^,^13^,^14^,^15^. In addition, they increasingly interact with invasive species, especially algae^6^,^16^,^17^,^18^. The responses of scleractinian corals to warming and the invasion of algal species have been reported separately. However, there is a lack of studies assessing the potential synergistic effects of both factors.

Presently, mortality events related to warming have affected several temperate Mediterranean anthozoan species^5^,^13^,^14^,^15^,^19^,^20^,^21^,^22^ and show contrasting levels of mortality at different spatial scales. Despite the broad geographical range of these mortalities, thermal adaptation in temperate Mediterranean corals and gorgonians, living in warmer vs. colder environments, has been explored in only a few studies and with inconclusive results^23^,^24^,^25^,^26^. In addition, there is a lack of studies on thermal adaptation in relation to differing geographical temperature regimes in temperate scleractinian reef-builders.

In relation to biological invasions, the Mediterranean Sea is a hot-spot for non-indigenous species, hosting almost 1,000 introduced species of which 128 are macrophytes^27^. In addition, experimental studies have shown the potential negative impacts of invasive algae on Mediterranean anthozoan species^28^.

Disentangling the relative importance of the different factors behind the responses of a species is the key to better foresee the potential effects of global change. In this study, we explore the potential local adaptations to temperature conditions and the synergistic effect of warming and invasive species. We focus our study on the Mediterranean endemic reef-builder *Cladocora caespitosa* because it is a good example of the abovementioned problems, being currently exposed to both stressors: warming and invasive algal species. First, we evaluated the potential adaptation to thermal stress by studying two populations subjected to contrasting thermal and necrosis histories with the aim of covering a broader and more representative range of potential responses in relation to thermotolerance; second, we assessed the effects of both warming and the presence of invasive algae covering the polyps to detect potential synergies that may negatively affect the species. Information on potential synergies between global change-related stressors that are overlapping geographically is critical to evaluate further threats to this and other similar endangered species.

## Results

### Temperature regimes and *in situ* necrosis

**Figure 1:**
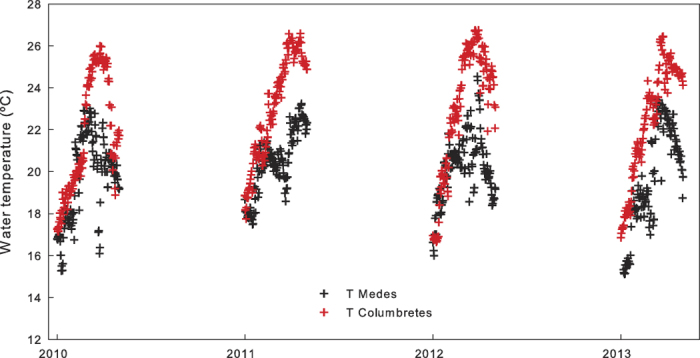
Summer temperature (June - September) at 15 m depth in the Columbretes and Medes Islands.

The sea water temperature profile at 15 m in the Columbretes and Medes Islands showed contrasting conditions during the studied summers (2010 – 2013). During these years, the temperature at 15 m reached maxima of 27.7 °C and 24.8 °C in the Columbretes and Medes Islands, respectively, and the mean summer temperature was 2 – 2.7 °C higher in the Columbretes than in the Medes Islands (Fig. 1).

While the *C. caespitosa* population of the Columbretes Islands has suffered repeatedly from warming-related necrosis events (including after the summers of 2010, 2011 and 2012^5^), the 4 year monitoring of the coral colonies in the Medes Islands showed no signs of mortality; neither after the summers of 2010 - 2013, nor in the recent past, as necrosis effects are detectable on colonies many years after mortality events.

### Thermotolerance experiment (T1)

**Figure 2:**
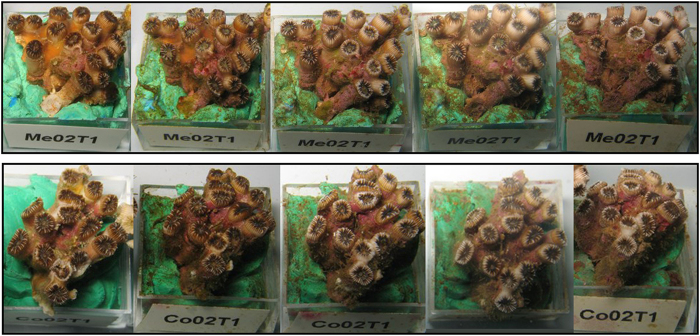
Photographs of the same nubbin throughout the thermotolerance experiment (T1) taken in ~2 week intervals. First row: nubbin from the Medes Islands. Second row: nubbin from the Columbretes Islands.

During the aquaria experiment, no signs of tissue necrosis (or bleaching, i.e., massive zooxanthellae loss) were observed on the *C. caespitosa* colonies from both Medes and Columbretes Islands populations (including the control) subjected to the temperature treatment (Fig. 2).

**Figure 3:**
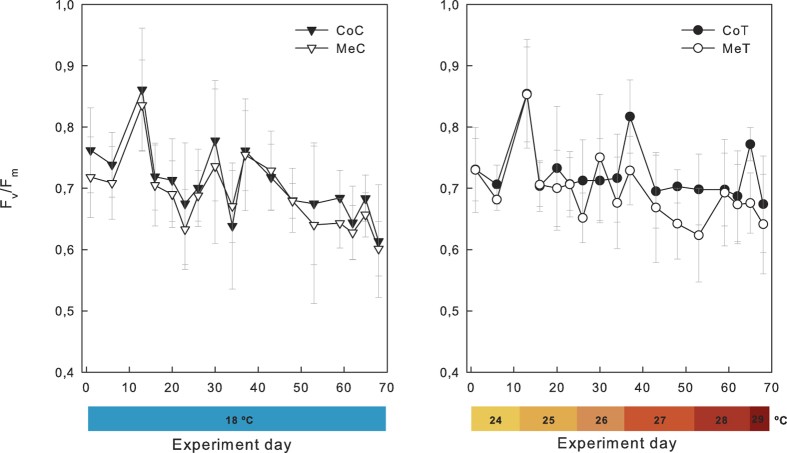
Maximum photosynthetic efficiency (±SD) in *Cladocora caespitosa* polyps from the Columbretes and Medes Islands subjected to control and temperature treatment (T1). N = 15 nubbins per population and treatment (see replication details in the methods section).

No significant differences in maximum photosynthetic efficiency (F_v_/F_m_) were found in the interaction between the location (Medes and Columbretes Islands) and the treatment (control and temperature increase) (F_1,908_ = 0.94, p = 0.33) (Fig. 3).

### Synergistic interaction between temperature and invasive alga (T2)

**Figure 4:**
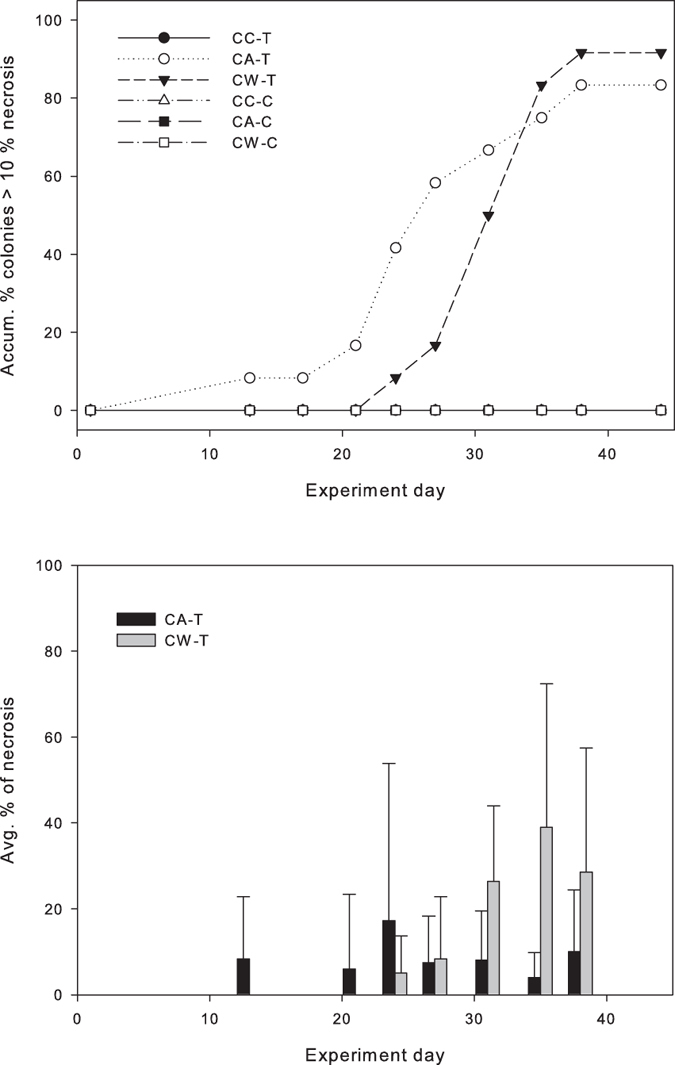
(Top) Accumulated percentage of colony fragments with necrosis rates over 10%. Subjected to thermal stress and invasion treatments [with invasive algae (CW-T), artificial material CA-T) and uncovered colonies (CC-T)] and subjected to temperature control and invasion treatments [with invasive algae (CW-C), artificial material CA-C) and uncovered colonies (CC-C)]. (Bottom) Average percentage of necrosis (±SD) in the CA-T and CW-T treatments. N = 12 nubbins per treatment (see replication details in the methods section).

For the second experiment, significant differences in necrosis were found between the temperature treatment (F_1,635_ = 23.33, p < 0.0001) and the invasive algae treatments (F_2,635_ = 10.38, p < 0.0001), which showed a significant interaction (F_2, 635_ = 9.00, p < 0.0001). Colonies subjected simultaneously to temperature and *Womersleyella setacea* overgrowth (CW-T) or artificial material treatments (CA-T) were severely affected by necrosis, especially during the second half of the experiment. At the end of the experiment, 91.7% and 83.3% of the colonies subjected to the alga and artificial material treatments, respectively, were affected to some extent by necrosis (over 10% of the colony area was necrosed). Maximum average necrosis reached 39% and 17% during the experiment in the alga and artificial treatments, respectively (Fig. 4). In contrast, all of the colonies in the temperature control tanks and those subjected only to the temperature treatment (CC-T) showed no necrosis signs over time (Fig. 4).

**Figure 5:**
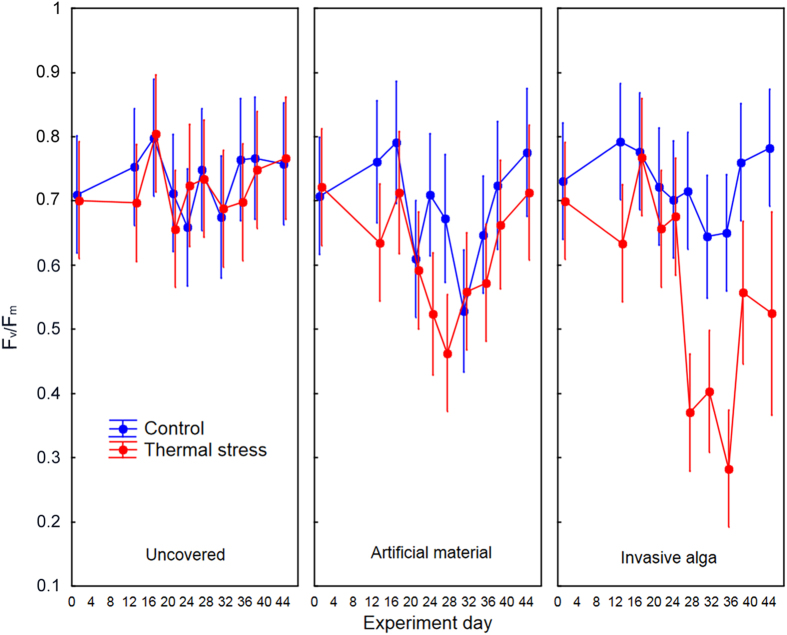
Maximum photosynthetic efficiency (±SD) in *Cladocora caespitosa* colonies subjected to control, thermal stress and three treatments of invasion: uncovered, with artificial material and with invasive algae. N = 12 nubbins per treatment (see replication details in the methods section).

Maximum photosynthetic efficiency (F_v_/F_m_) showed significant differences between temperature treatments (F_1, 605_ = 42.95, p < 0.0001), invasive algae treatments (F_2, 605_ = 14.12, p < 0.0001), and their interaction (F_2, 605_ = 12.15, p < 0.0001); overall, there was an average decrease of 15.6% in the temperature plus artificial material treatment and of 24.1% in the temperature plus alga treatment throughout the experiment, with a partial recovery at the end (Fig. 5). Again, all of the colonies in the temperature control tank and those subjected only to the temperature treatment (CC-T) showed no significant decrease in photosynthetic efficiency (Fig. 5).

## Discussion

Understanding how global change-related disturbances interact is critical in the current context of rapid changes and the enlargement of the geographical overlap of stressors. Using an experimental approach, we showed that the interaction of two of the most widespread global change stressors (warming and invasive species) trigger drastic additive effects on a temperate reef-builder coral that shows uniform thermotolerance despite contrasting temperature regimes inherent to its geographical distribution.

While mortalities of *Cladocora caespitosa*, related to abnormally high summer temperatures, have been reported in many Mediterranean coastal zones exposed to differing thermal regimes (La Spezia, NW Italy^13^; Columbretes, E Spain^5^; Mljet and Piran, Croatia and Slovenia^15^; Cyprus^14^), information on its thermotolerance in relation to geographical differences in temperature is lacking. Strikingly, our results showed that *C. caespitosa* colonies from a population recurrently affected by positive thermal anomalies and mortality events (Columbretes Islands^5^) and colonies thriving in much colder waters and at least not recently impacted by mortality events (Medes Islands: this study), did not exhibit signs of necrosis after being exposed over 68 days to thermal stress conditions. In the present study, temperatures in the aquaria experiment were similar or even higher to those recorded in summers triggering necrosis in the Columbretes Islands^5^, and the maxima were over 4 °C higher than those registered in the Medes Islands. However, in contrast to what has been described *in situ*^5^ or in previous experimental studies^29^,^30^,^31^, no significant effects on necrosis and photosynthetic efficiency were recorded. Previous studies on temperate octocorals determined similar discrepancies between experimental and *in situ* studies^23^,^24^,^26^. These contrasting results support the hypothesis of the multi-factorial cause of the necrosis events affecting *C. caespitosa*^5^ or similar species^32^. This shows that this species, despite its potential broad thermal tolerance in the aquaria, is subjected to unstudied factors and synergies that need further investigation.

Overall, the results of the thermotolerance experiment are noteworthy, considering that *C. caespitosa* is an endemic species that has been thriving in the Mediterranean Sea for the last 2.5 million years (Late Pliocene^33^) and with populations showing a certain grade of isolation and genetic differentiation^34^. Therefore, hypothetically, local thermal adaptation could be expected as described in other Mediterranean and tropical benthic species^24^,^25^,^35^,^36^. However, our results are similar to those obtained for the Mediterranean sleractinian coral *Oculina patagonica*^32^, which belongs to the same family as *C. caespitosa*^37^. These contrasting results raise further questions on the potential for thermal adaptation in temperate corals and further studies are needed to understand these processes.

While neither necrosis nor a strong reduction of photosynthetic efficiency was registered during the thermotolerance experiment, both variables were significantly affected when subjected to both a temperature increase and invasive algae (Figs 4 and 5). However, it has to be noted that photosynthetic efficiency showed a partial recovery at the end of the experiment in the covered treatments, both under control and enhanced temperature. This recovery could be associated to a potential low-light photo-acclimation mechanism of the zooxanthellae in symbiosis with *C. caespitosa*, as has been reported to occur after some days in tropical corals^38^ and in agreement with the capability of *C. caespitosa* of dwelling in contrasting light conditions^39^.

Both the invasive algae and the artificial material treatments had negative effects on the coral, which could point to a primary influence of a physical effect of overgrowth. However, although secondary metabolites of *W. setacea* have not been studied to date, chemical interactions should not be disregarded, taking into account that comparatively the alga treatment had increased negative effects, both in tissue necrosis and photosynthetic activity.

These types of interactions have been suggested in tropical reefs between macroalgae and corals^40^ or related to algae-associated pathogens^41^. It should be noted that turf algae have been reported to notably overgrow and damage coral tissue in tropical corals. For example, the filamentous red alga *Anotrichium tenue* was reported to damage *Porites spp*. and outcompete coral colonies through physical and chemical mechanisms^42^. In our study, besides potential chemical interactions, thermal stress was exacerbated by the physical shield effect of *W. setacea* directly covering the colonies. As mentioned in previous studies on sponges, this effect reduces or even impairs water exchange with the associated impact on respiratory and nutritional needs^43^. Hence, similar effects on the physiological status of *C. caespitosa* colonies can also be expected because its nutrition relies on both autotrophy and heterotrophy, thus being highly dependent of water exchange and light availability^44^.

Recently, several studies have underlined the crucial need to account for multiple-stressor interactions in ecological studies and conservation planning^7^,^9^,^10^. The results reported in our study on the synergistic effects between warming and invasive species are worrying, since impacts on *C. caespitosa*, in particular, and other macroinvertebrates, in general, could notably increase when considering future warming and the spread of invasive species in the Mediterranean region.

## Methods

### Cladocora caespitosa

*Cladocora caespitosa* is a long-lived, slow-growing coral^39^ with parsimonious dynamics and therefore limited capacity to recover from catastrophic disturbances^45^. This species has been repeatedly impacted by necrosis events that are primarily related to warming^5^,^13^,^14^,^15^, and the response of *C. caespitosa* to warming has been studied in both the laboratory^29^,^30^ and *in situ*^5^,^15^. However, although it is a widely distributed species throughout the Mediterranean, its ability to adapt to different thermal regimes is still unknown. In addition, its habitat has been reported to be invaded by at least two invasive algae so far (*Lophocladia lallemandii* and *Caulerpa cylindracea*^3^,^6^), and due to its relatively broad depth distribution range, it is potentially exposed to many other species.

### *In situ* necrosis and thermal history

**Figure 6:**
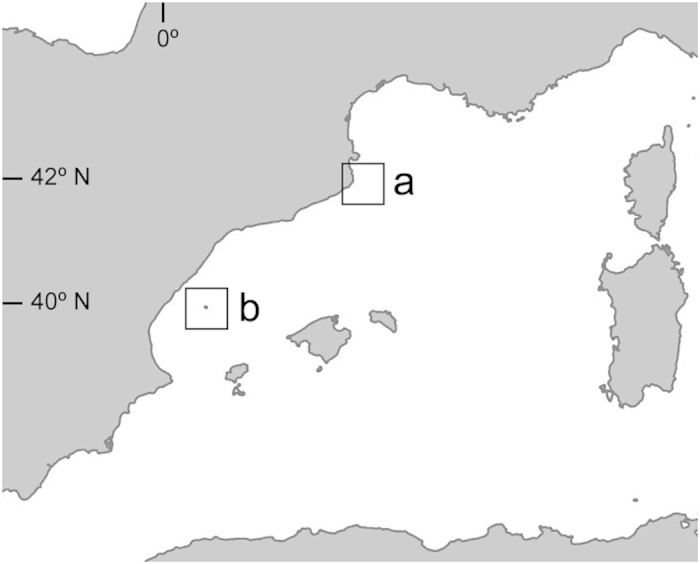
Western Mediterranean Sea. Medes Islands (**a**) and Columbretes Islands (**b**). Map created using Adobe Photoshop 8.0 (http://www.photoshop.com).

With the objective of comparing *C. caespitosa* populations with contrasting necrosis and thermal histories, previous information on temperature regimes and mortality incidence was obtained in two distant NW Mediterranean marine protected areas, where populations are naturally subject to highly contrasting thermal regimes: the Medes and Columbretes Islands (Fig. 6).

The *C. caespitosa* population in the Columbretes Islands is known to have suffered recurrent warming-induced necrosis events^5^. For the present study, information on the occurrence of warming-induced necrosis events in the population of the Medes Islands was obtained through the monitoring of 30 colonies from 2010 to 2013 and following the same methodology used in the Columbretes Islands population^5^. Moreover, the absence of necrosis in the Medes Islands area was also noted during regular monitoring activities on other species that were conducted during the previous decades.

To search for differences in the thermal regimes between both sites, hourly temperature data were recorded from dataloggers (Water Temp pro v2, ONSET, Cape Cod, MA, USA) installed at 15 m depth (average depth were the coral colonies occur) at each location. Because warming-related necrosis in this coral occurs directly after the summer^5^, only the summer water temperature data were analyzed (June - September from 2010 to 2013).

### Sample collection and aquaria set-up

Healthy colonies of *Cladocora caespitosa* were sampled in the Medes and Columbretes Islands. At each site, ~30 differentiated colonies were sampled during the summer of 2011 and transported in aerated seawater to the Experimental Aquarium Facilities of the Institute of Marine Sciences in Barcelona in less than 24 h.

Upon arrival to the facilities, the colonies were installed in aquaria continuously supplied with seawater (salinity 38%). Each tank had an inlet pipe for the supply of fresh seawater and an outlet pipe, which remained open so that the tanks functioned as an open system. All of the experiments used the same two aquarium settings: the control and a treatment group with three replicas per setting. In the temperature treatment, the seawater was heated in a buffer tank equipped with submersible resistance heaters and regulated by temperature controllers (Aqua Medic T controller). The experimental tanks were equipped with submersible pumps to facilitate water circulation. The submersible pumps provided a continuous circular current with a flow rate of approximately 60 l h^−1^. The colonies were fed a supply of ocean nutrition^TM^ that included frozen Cyclops alternated with a supply of “Benthic nutrition” prepared with the aquarium food mixture.

The irradiance was obtained from 30 W cool white fluorescent bulbs, which used an on/off regime of a 12 h light-dark cycle and was calibrated to match the light intensity of both populations that received, in their natural environment, approximately 50 to 150 μmol photons m^−2^ s^−1^ (authors’ unpublished data).

### Aquaria experimental design

A thermotolerance experiment (hereafter, T1) was run with colonies from both populations (Columbretes and Medes Islands) to test for differences in the response to warming of the colonies thriving in contrasting thermal regimes. The second experiment (hereafter, T2) was conducted to search for potential synergistic effects of both warming and invasive algae on the coral colonies. Only colonies from the Medes Islands population were used to run experiment T2. Both experiments were conducted from September to November 2011.

For the first comparative thermotolerance assessment (T1), 15 of 30 nubbins (each nubbin was a colony fragment of 5–10 polyps; hereafter colony) from each population were placed in each treatment. Each treatment was composed of three tanks (approximately 48 l), where 5 nubbins from each population were placed and subjected to the following temperature treatment: 11 days at 24 °C, 14 days at 25 °C, 11 days at 26 °C, 17 days at 27 °C, 11 days at 28 °C and 4 days at 29 °C. The time interval at each temperature was set in accordance to the thermal regime registered in the summers with *C. caespitosa* mortality events^5^. Another set of 15 colony fragments of each population were kept as controls in a second set of aquaria at a constant temperature of 17 °C.

The second experimental set-up (T2) consisted of a similar temperature and control treatment that was kept for 44 days at 25 °C and 17 °C, respectively, with three aquaria per treatment. In each aquarium, 3 sets of 4 nubbins (5–10 polyps each) were subjected to the following invasive algae treatment: 1 set was completely covered by *Womersleyella setacea* (CW), 1 set was completely covered by an artificial plastic material (CA) and the last set was left uncovered (CC). In the covered treatments both the alga and the plastic sponge-like material (both circle shaped of about 7–8 cm in diameter and 1–2 cm thick) were hold by gravity in direct contact with the nubbins, simulating the way *W. setacea* turf covers substrata in natural environments. The inert artificial material (dark-colored plastic sponge) was chosen to simulate the physical effect of turf algal cover. The invasive alga *Wormersleyella setacea* was chosen as a model turf algal species because it exhibits the most problematic traits in relation to the potential impact to benthic communities; i.e., it forms persistent carpets that completely cover rocky substrata and it is perennial, showing little or no seasonality^4^. In addition, *W. setacea* is currently starting to overlap its distribution with that of *C. caespitosa* in circalitoral assemblages (e.g., the Medes Islands, authors’ pers. obs.).

The response variables measured in both experimental set-ups (T1 and T2) were the necrosis and photosynthetic fitness of each colony. The necrosed colony area was recorded, both visually and photographically, at regular intervals (3–6 days). Necrosis rates were estimated in 10% intervals^5^, and the colonies were registered as affected when exceeding 10% of the necrosed area. Thermal stress in corals is readily detectable as a decrease in the photosynthetic fitness of the zooxanthellae using non-invasive pulse amplitude modulation (PAM) fluorometry^46^. The maximum photosynthetic efficiency (F_v_/F_m_) was measured using an underwater Pulse Amplitude Modulated fluorometer (Diving-PAM, Walz, Effeltrich, Germany). Before measurements, all nubbins were kept in the dark during 1 hour to ensure a dark-adapted state of all specimens. The maximum quantum yield was measured by exposing the polyps to a 0.8 seconds period at saturating light (>8,000 μmol photon m^−2^ s^−1^). Measurements were always undertaken in polyp tissues without signs of necrosis.

### Statistical analysis

Linear mixed effects models (nlme package for R^47^) were used to test for differences in necrosis rates and photosynthetic efficiency between both studied populations (experiment T1) and algal-temperature synergistic effects (experiment T2). Linear mixed effects models are used to model data that can cope with repeated measures over time^47^. The fixed factors in experiment T1 were location (Medes and Columbretes Islands), temperature (warming and control) and time. In experiment T2, the fixed factors were invasive alga treatment (uncovered, covered with artificial material and covered with *W. setacea*), temperature (warming and control) and time. In both experiments, the tank was set as a random factor, and the response variables were the percentage of tissue necrosis and photosynthetic efficiency. The necrosis data were logit transformed. All of the analyses were computed using R^48^.

## Additional Information

**How to cite this article**: Kersting, D. K. *et al.* Experimental evidence of the synergistic effects of warming and invasive algae on a temperate reef-builder coral. *Sci. Rep.* **5**, 18635; doi: 10.1038/srep18635 (2015).
